# Lithium Monotherapy Increases ACTH and Cortisol Response in the Dex/CRH Test in Unipolar Depressed Subjects. A Study with 30 Treatment-Naive Patients

**DOI:** 10.1371/journal.pone.0027613

**Published:** 2011-11-23

**Authors:** Tom Bschor, Dirk Ritter, Patricia Winkelmann, Sebastian Erbe, Manfred Uhr, Marcus Ising, Ute Lewitzka

**Affiliations:** 1 Department of Psychiatry, Schlosspark-Clinic, Berlin, Germany; 2 Department of Psychiatry, Technical University of Dresden, University Hospital, Dresden, Germany; 3 Max Planck Institute of Psychiatry, Munich, Germany; 4 IWK Health Centre/Dalhousie University, Mood Disorder Research Clinic, Halifax, Canada; 5 International Group for the Study of Lithium Treated Patients (IGSLI), www.igsli.org; University of Adelaide, Australia

## Abstract

**Background:**

Distorted activity of the hypothalamic-pituitary-adrenocortical (HPA) system is one of the most robustly documented biological abnormalities in major depression. Lithium is central to the treatment of affective disorders, but little is known about its effects on the HPA system of depressed subjects.

**Objective:**

To assess the effects of lithium monotherapy on the HPA system of patients with major depression by means of the combined DEX/CRH test.

**Method:**

Thirty drug-naive outpatients with major depression (single episode or unipolar recurrent; SCID I- and II-confirmed) were treated with lithium monotherapy for four weeks. The DEX/CRH test was conducted directly before intake of the first lithium tablet and four weeks thereafter. Weekly ratings with the HDRS_21_ were used to determine response (≥50% symptom reduction) and remission (HDRS ≤7).

**Results:**

Lithium levels within the therapeutic range were achieved rapidly. Tolerability was good; no patient terminated the treatment prematurely. Response and remission rates were 50% and 33% respectively. Compared to the DEX/CRH test before the start of the treatment, a considerable and significant increase in all CRH-stimulated ACTH and cortisol parameters could be detected in the second DEX/CRH test. When analysed with particular regard to responders and non-responders, that significant increase was only present in the responders.

**Conclusions:**

We were able to demonstrate that lithium leads to a significant activation of the HPA system. This is possibly connected to stimulation of hypothalamic arginine vasoporessin (AVP), to direct intracellular effects of lithium on pituitary cells and to an induction of gene expression.

**Trial Registration:**

drks-nue.uniklinik-freiburg.de DRKS00003185

## Introduction

Depression is a frequent and severe medical problem. One of the cornerstones of treatment is antidepressant medication [1], [2]. However, a challenging issue in the pharmacotherapy of depression remains the high rate of non-response to antidepressants [3]. Therefore, a major aim of research is to develop alternative drugs with alternative modes of action.

A system repeatedly studied as a target for new antidepressive drugs is the hypothalamic-pituitary-adrenocortical (HPA) system [4], [5], [6]. It has been postulated that depression (or at least a subgroup of depression) is associated with overactivity of the HPA system, possibly due to impaired negative feedback control [7]. Most antidepressants are supposed to partly act by improving the negative feedback control [8]. Other substances reducing the overload or improving the negative feedback have been tested for the treatment of depression, however with ambiguous results [9], [10].

Another fundamental drug used to treat affective disorders with an alternative mode of action is lithium. Lithium induces multiple *intra*cellular modifications, which are yet not fully understood [11], [12], [13]. Beside its widespread use in the prophylactic treatment of bipolar [14], [15], [16] and unipolar affective disorder [1], [2], [17], lithium in the form of lithium augmentation is a major strategy for treating (unipolar) depression not responding to antidepressant monotherapy [18], [19].

Not established, though demonstrated effective in several older studies, is lithium monotherapy for the treatment of acute depression. At least seven studies from the 1970s and 1980s [20], [21], [22], [23], [24], [25], [26] showed that lithium monotherapy is as effective as a tricyclic antidepressant and the two of those studies that included a placebo component [21], [23] demonstrated superiority over the placebo (overview: [27]). Several of these older studies have methodological shortcomings (e. g. small sample size) [27]. To our knowledge, in the last 20 years lithium monotherapy for acute unipolar depression has not been the subject of systematic research.

The impact of lithium on the HPA system has repeatedly been investigated mainly in pre-clinical studies. Older cell culture and animal studies [28], [29], [30], [31], [32], [33], [34] showed an enhancing effect of lithium on the release of CRH, AVP, ACHT, and cortisol, and on the glucocorticoid receptor (GR) messenger RNA. With regard to humans, certain older studies with methodological limitations showed an increase of plasma cortisol in affectively ill patients after initiation of lithium therapy [35], [36], whilst other did not [37]. After long-term lithium treatment, the findings have tended to show a decrease in plasma cortisol levels [38].

In a previous study, we investigated a group of unipolar depressed patients (N = 30) who did not respond to a treatment with an antidepressant and who were subsequently treated with lithium augmentation [39], [40]. We conducted the most sensitive challenge test of the HPA system, the combined dexamethasone/CRH test (DEX/CRH test) [8], [41], right before, and two to four weeks during, lithium augmentation. Interestingly, patients had a significantly higher ACTH and cortisol response to CRH stimulation in the DEX/CRH test during lithium augmentation compared to the values at baseline, regardless of response to the lithium augmentation [39], [42].

Because it couldn't be concluded from the results of this preceding study whether the increase in HPA system activity was due to a direct pharmacological effect of the lithium ion or whether it was instead an effect of the complex lithium augmentation mechanism (i. e. pre-treatment with an antidepressant, followed by a lithium/antidepressant combination), we here present the results of a study with 30 unipolar, acutely depressed, drug-naive subjects who were treated with lithium monotherapy.

## Methods

The protocol for this trial and supporting CONSORT checklist are available as supporting information; see Checklist S1 and Protocol S1.

This study was conducted at the Department of Psychiatry, Technical University of Dresden between January 2003 and February 2004. Patients who contacted the outpatient clinic of the department on their own, were transferred to the outpatient clinic by other physicians, or who followed a call for study participation in the local newspapers could be included into the study. Except for one patient, who was transferred to the inpatient ward because of the severity of the symptoms, all participants were outpatients. The study protocol was approved by the local ethical committee and the study has been carried out in accordance with the Declaration of Helsinki. After complete description of the study to the subjects, written informed consent was obtained. The laboratory ACTH and cortisol measurements and the statistical analyses were performed at the Max-Planck Institute of Psychiatry, Munich.

### Inclusion criteria

Patients of both genders, aged 18 to 75 years, with a major depressive episode, single episode or recurrent (DSM IV criteria) were included into the study. Diagnoses were confirmed by the Structured Clinical Interview for DSM IV (SCID I; German version) [43], [44], [45]. On the day of study, included participants had to have a score of at least 15 on the Hamilton Depression Rating Scale, 21-item version (HDRS_21_) [46]. Due to the inclusion criteria participants had to have been free of psychotropic drugs for at least 14 days (fluoxetine: 35 days) with the exception of benzodiazepines (max. 20 mg diazepam or equivalent per day). However, we were able to include only subjects who have not been treated pharmacologically for the index episode (only drug-naïve patients).

### Exclusion criteria

The exclusion criteria were (SCID I): a history of a manic or hypomanic episode (bipolar disorder), current alcohol abuse or dependency, organic brain disease, schizophrenia or schizoaffective disorder. Further exclusion criteria were: current intake of psychotropic drugs (see above); acute or chronic somatic conditions that might contraindicate lithium or that might influence the regulation of mineralo- or glucocorticoids; pregnancy or lactation. Personality disorders were not an exclusion criterion but were systematically recorded by conducting a SCID II interview with each subject.

### Lithium treatment

Patients started lithium treatment on the evening of the first combined DEX/CRH test (baseline). Lithium was administered at a daily dose of two times 12.2 mEq lithium carbonate (2×450 mg). Standardized 12-hour serum lithium levels were measured at day 3, 7, 14, 21, and 28 of lithium treatment. The lithium dose was adapted individually to achieve a serum level within the established therapeutic range [47] between 0.5 and 1.0 mEq/L.

### Psychopathological ratings, response and remission criteria

The HDRS_21_, the Clinical Global Impressions Scale (CGI) [48] and the Beck Depression Inventory (BDI) [49] used as a self-rating scale were conducted at weekly intervals (on day 0, 7, 14, 21, 28) by trained and experienced raters (TB, DR, or UL). In accordance with established criteria [50], [51], patients were classified as responders if their HDRS_21_-score showed a decrease of 50% or more in the course of the four-week-study and remission was defined as a HDRS_21_-score of 7 or less on day 28. Response or remission had to be confirmed after one week by the same criteria. In line with most studies on antidepressive drugs we decided to use the HDRS_21_ to define response and remission, because it is a rater based scale (in contrast to the BDI) and it is much more sophisticated as compared to the one-item CGI.

### The Dexamethasone/CRH test

The combined DEX/CRH test was performed in accordance with the established research procedure [6], [41], [52], [53], [54]: The patients were pre-treated with an oral dose of 1.5 mg of dexamethasone at 11 p.m. The next day, blood was drawn at 3 p.m., 3.30 p.m., 3.45 p.m., 4 p.m., 4.15 p.m., and 4.30 p.m. through an intravenous catheter (through the wall technique). 100 µg of human CRH was administered intravenously at 3 p.m. shortly after the first blood collection. All blood samples were collected in pre-frozen, EDTA- and aprotinin (Trasylol™)-containing tubes and immediately centrifuged in a centrifuge cooled to 4°C. The plasma was frozen directly after separation from the blood cells and stored at −80°C.

The plasma cortisol and ACTH concentrations in the first specimen (baseline), collected at 3 p.m., reflect the suppressive effects of the dexamethasone administered the day before, whereas the other five plasma cortisol and ACTH concentrations reflect the additional effects of the CRH stimulation. Peak levels, CRH response values (Delta) and area under the curve (AUC) values of the cortisol and ACTH in the six consecutive blood samples were used as indicators of the response to the combined DEX/CRH test. The Delta values were obtained by subtracting the 3 p.m. hormone values (before CRH injection) from the individual peak value. The AUC values were defined as the natural logarithms of the trapezoidal integration of the six cortisol and ACTH concentrations. Missing values (about 1%) were estimated by the individual values of the previous and subsequent sample.

### Laboratory analysis

All probes were analysed together in one assay at the end of the entire study. In order to determine the plasma cortisol concentration, a commercially available radioimmunoassay (RIA) kit (ICN Biomedicals, Carson, CA) was used. The detection limit was 0.3 ng/ml plasma; intra- and interassay coefficients of variation for 20 and 40 ng/ml were <7%. For plasma ACTH measurements, an immunoradiometric assay without extraction (Nichols Institute, San Juan Capistrano, CA) was used providing a detection limit of 4.0 pg/ml. The intra- and interassay coefficients of variation at 20 pg/ml plasma were <8%.

### Sample size calculation

The study was designed according to the above mentioned previous results from our group [39]. According to these findings, we expected an effectsize d(z) ( = Cohen's d for dependent measures) of 0.65 for observing an increased cortisol response (AUC) to a second DEX/CRH test after four weeks of lithium treatment. From this effect size, we calculated an optimal sample size of n = 29 under standard assumptions (alpha error = 0.05; power = 0.9) using G*Power 3.1 [55].

### Statistical Analysis

Changes between the initial clinical or endocrine variables at baseline and at follow-up were evaluated with Wilcoxon's matched pairs test. Differences between responders and non-responders were assessed with Fisher's exact test and the Mann-Whitney U test. Differences were regarded as significant when p<0.05. All analyses were performed with SPSS, Version 17.0. The sample size estimation was performed with G*Power 3.1 [55].

## Results

### Study population

**Figure 1:**
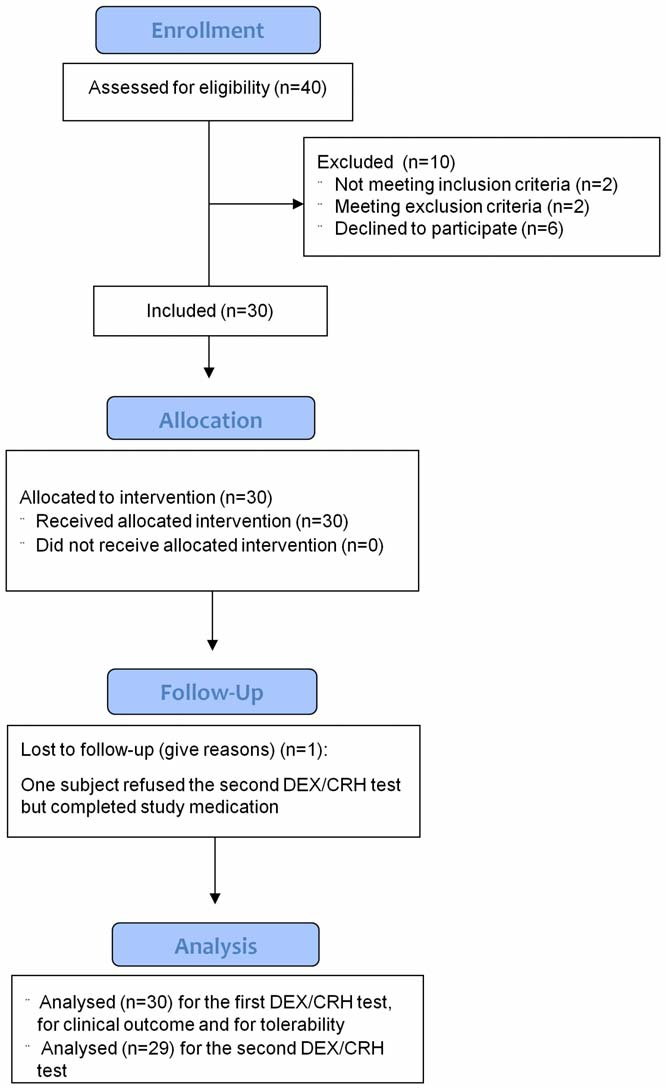
CONSORT 2010 Flow Diagram.

Forty patients were screened for participation and 30 were able to be included in the study (Figure 1). Reasons for non-inclusion were: no diagnosis of depression (N = 2), significant somatic comorbidity (N = 2), refusal to participate (N = 2), and non-appearance at the first study visit (N = 3). One patient refused lithium treatment after having accepted the baseline DEX/CRH test. She was excluded from the study. The remaining 30 patients (15 men, 15 women) completed four weeks of lithium treatment but one patient (male) refused the second DEX/CRH test. The HDRS_21_ score was 21.7±4.7 (mean ± SD, range: 15–33) at study entry. For details on demographic and clinical data see Table 1.

**Table 1:** Demographic and clinical data of the study population (N = 30) (mean ± standard deviation).

Age (years)	45.97±10.93
Gender (male:female)	15∶15
Age at onset of mood disorder (years)	36.83±10.24
Duration of index episode (months)	17.03±16.26
Number of previous depressive episodes	1.77±1.78
diagnosis: single episode (DSM-IV: 296.2) : recurrent episodes (DSM-IV: 296.3)	9∶21

### Treatment and clinical outcome

Lithium serum levels and lithium doses are presented in Table 2.

**Table 2:** Lithium serum levels and lithium doses (N = 30).

	lithium carbonate doses		Lithium serum level (mEq/L)	
	mean ± SD	Range	mean ± SD	range
day 3, mEq/d	24.4±0	–	0.46±0.11	0.26–0.69
mg/d	900±0	–		
day 7, mEq/d	26.84±4.96	18.3–36.6	0.56±0.14	0.26–0.88
mg/d	990±183	675–1350		
day 14, mEq/d	33.55±6.94	18.3–48.8	0.71±0.19	0.45–1.31
mg/d	1238±256	675–1800		
day 21, mEq/d	35.99±8.68	18.3–48.8	0.81±0.18*	0.51–1.38*
mg/d	1327±320	675–1800		
day 28, mEq/d	35.99±9.21	18.3–48.8	0.82±0.19	0.45–1.39
mg/d	1327±340	675–1800		

*the data from two patients are missing.

Lithium tolerability was good. Side effects were recorded weekly. The majority of patients actually showed signs of probably side-effects of lithium (70% of the patients at day 28), however such side-effects were generally mild. No patient terminated the lithium treatment prematurely. The side-effects observed were those that are typically expected under lithium treatment (in order of appearance: polyuria, ataxia, increased thirst, diarrhoea, and tremor).

According to the criteria mentioned above, fifteen of the 30 patients (8 women, 7 men) responded within 4 weeks after initiation of lithium treatment (in mean after 2.33±1.18 weeks). Ten of these 15 responders even showed remission. There was no significant difference between responders and non-responders with regard to age, gender, baseline severity of depression, lithium doses or lithium serum level (data not shown). The HDRS_21_, BDI and CGI_severity_ scores (mean ± SD) on day 0 were 21.7±4.70, 24.0±8.1, and 5.7±0.7 respectively, and declined to 11.1±5.1, 15.0±9.1, and 4.6±1.2 on day 28 (p<0.001 for all three changes; Wilcoxon's matched pairs test).

### Endocrinological results

#### Full group

At day 0 (before start of lithium treatment), the ACTH peak was 25.37±27.19 pg/ml and the cortisol peak was 83.77±81.49 ng/ml (for baseline, AUC, and delta values, and cortisol/ACTH ratios cf. Table 3). After four weeks of lithium treatment (day 28) ACTH at baseline (before CRH injection) was comparable to day 0, but both all CRH-stimulated ACTH parameters and all cortisol parameters were significantly higher during lithium treatment compared to pre-treatment (ACTH peak 28.48±24.65 pg/ml, p = 0.048; cortisol peak 104.84±78.95 ng/ml, p = 0.012; cf. Table 3).

**Table 3:** Endocrinological results of the combined dexamethasone/CRH test before and after four weeks of lithium monotherapy in 30 unipolar depressed patients (mean, SD).

		pre-treatment (day 0) (N = 30)	after 4 weeks of lithium (day 28) (N = 29)	pWilcoxon's matched pairs test
**ACTH** (pg/ml)	baseline (before CRH injection)	16.40±7.60	16.25±6.47	0.991
	ACTH peak	25.37±27.19	28.48±24.65	0.048
	ACTH AUC	1829.3±1488.9	1978.59±1129.47	0.033
	ACTH delta	8.97±26.20	12.23±19.74	0.008
**Cortisol** (ng/ml)	baseline (before CRH injection)	17.28±13.40	28.34±35.99	0.002
	Cortisol peak (ng/ml)	83.77±81.49	104.84±78.95	0.012
	Cortisol AUC	4933.6±4640.2	5927.76±4323.2	0.013
	Cortisol delta	66.48±73.51	76.5±52.46	0.045

#### Responder and non-responder

With regard to response or non-response to the lithium treatment, differences could be detected between responders and non-responders. Before the start of the treatment (day 0), patients who responded in the following four weeks to the lithium medication displayed numerically lower values on all ACTH and cortisol parameters than subsequent non-responders. However, only the difference for baseline ACTH (before CRH injection) was significant (14.16±1.85 vs. 18.64±10.27 pg/ml, p = 0.049, Mann-Whitney U test, cf. Table 4).

**Table 4:** Results of the combined dexamethasone/CRH test in unipolar depressed responders and non-responders to a four week lithium monotherapy (mean ± SD).

		Pre-treatment (day 0)			after 4 weeks of lithium (day 28)			day 0 vs. day 28	
		Responders(n = 15)	Non-Responders(n = 15)	p(Responders vs. Non-Responders. Mann-Whitney U Test)	Responders(n = 14)	Non-Responders(n = 15)	p(Responders vs. Non-Responders. Mann-Whitney U Test)	Respondersp(Wilcoxon's matched pairs test)	Non-Respondersp(Wilcoxon's matched pairs test)
**ACTH** (pg/ml)	baseline (before CRH injection)	14.16±1.85	18.64±10.27	0.049	17.61±8.74	14.97±3.02	0.81	0.221	0.233
	ACTH peak	19.56±3.62	31.18±38.02	0.95	35.32±34.34	22.10±5.41	0.57	0.008	0.733
	ACTH AUC	1497.9±186.3	2160.8±2078.9	0.66	2308.4±1558.1	1670.8±294.7	0.66	0.013	0.609
	ACTH delta	5.41±4.32	12.54±37.10	0.08	17.71±27.20	7.13±5.84	0.16	0.035	0.084
**Cortisol** (ng/ml)	baseline (before CRH injection)	15.08±9.68	19.49±16.365	0.69	28.22±28.53	28.44±42.83	0.76	0.004	0.125
	Cortisol peak (ng/ml)	73.39±43.05	94.14±108.03	0.55	114.95±67.25	95.39±89.82	0.22	0.026	0.112
	Cortisol AUC	4231.1±2585.8	5394.8±6046.2	0.63	6564.2±3827.5	5333.8±4795.2	0.26	0.026	0.173
	Cortisol delta	58.31±44.01	74.65±95.46	0.74	86.73±51.51	66.95±53.27	0.27	0.041	0.334

**Figure 2:**
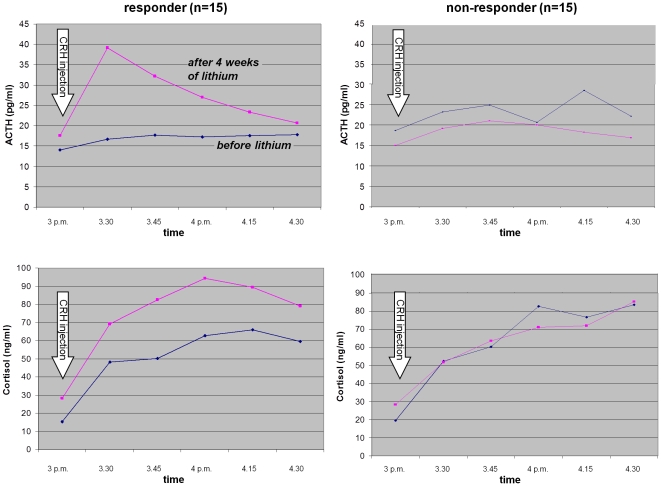
ACTH and cortisol response in the combined DEX/CRH test on the day before lithium monotherapy (blue line) and after four weeks of lithium treatment (pink line) in responders and non-responders (n = 29).

After four weeks of lithium treatment (day 28) a substantial rise in the ACTH and cortisol parameters (significant for all parameters except baseline ACTH) was observed only in responders to the lithium treatment, while the non-responders showed a (non-significant) decrease for most of the parameters of the DEX/CRH test (cf. Figure 2). After this adjustment (responders started with lower pre-treatment values but exhibited an increase, while non-responders started with higher pre-treatment values but tended to develop a decrease), no relevant differences in the DEX/CRH test results could be found between responders and non-responders at day 28.

## Discussion

This is, to our knowledge, the first systematic study of lithium monotherapy in treating acute unipolar depression in more than 20 years. Lithium serum levels can be considered as adequate and well-tailored, reaching the therapeutic range after just one week, with a low degree of variance. The response (50%) and remission (33%) rates have to be called moderate, but match the efficacy typically seen in clinical studies on antidepressive compounds. In a recent analysis including 182 RCTs of antidepressants, the pooled response rate was 53.8% [56]. However, this study was not designed to test the efficacy of lithium monotherapy. Because neuroendocrinology was the field of enquiry, no control group was included. Lithium tolerability was good, despite mean lithium serum levels of >0.8 mEq/L at week 3 and 4.

After 4 weeks of lithium treatment the HPA system showed a clear activation compared to the first DEX/CRH test directly before the start of lithium (p<0.05 for all ACTH and cortisol parameters after CRH stimulation). This is in line with previous results from pre-clinical studies and our study on lithium augmentation. Previous studies in cell cultures and in animals [28], [30], [31], [32] as well as in depressed subjects [35], [36] showed a stimulating effect of lithium on CRH, ACTH and cortisol. In addition, in a cross-sectional study with unipolar and bipolar patients in different mood states, Watson and colleagues found a significantly more pronounced cortisol response in the combined DEX/CRH test when comparing patients taking lithium with those not taking lithium [57]. However, ours is the first study demonstrating the HPA-activating effect of lithium monotherapy in unipolar depressed patients with the DEX/CRH test and a prospective design.

In an earlier study with the DEX/CRH test, our group found that lithium augmentation had a clear HPA-system-activating effect in unipolar depressed patients who did not respond to preceding monotherapy with an antidepressant [39], [40], [42]. Due to the study design it was not clear if the endocrinological changes observed were a direct effect of the lithium ion or if they required preceding treatment with an antidepressant. It is well known that most antidepressants lead to deactivation of the HPA system [7], [8], [58]. Given this fact, one explanation for the rise of the CRH-stimulated parameters in the DEX/CRH test during lithium augmentation could be that lithium augmentation only restores the endocrinological situation before the start of the antidepressant. Another explanation could be that the lithium's enhancing effect on the HPA system requires neurobiological changes made by preceding antidepressant treatment, e. g. a sensitisation of post-synaptic serotonin receptors [59], [60].

However, with the results of the presented study it becomes likely that it is a direct effect of lithium that activates the HPA system in such way that it becomes more responsive to CRH and ACTH and probably to external (stress) stimuli as well. A probable explanation of this finding is related to the osmotic effect of lithium salts. It is well known that lithium treatment affects renal tubular function and can lead to polyuria [61], [62]. As a compensatory function, synthesis and secretion of arginine vasopressin (AVP) from the paraventricular nuclei of the hypothalamus is stimulated. This was shown in rodents [63], whilst Watson et al. [57] reported consistent plasma AVP increases in lithium-treated patients with bipolar disorder or chronic depression. Previously, it has been shown that continuous infusion with vasopressin in healthy subjects stimulates the HPA axis and leads to increased ACTH and cortisol responses to the combined DEX/CRH test [64].

The hypothetical functional connection between elevated AVP and hyper-responsiveness of the HPA axis is further supported by animal studies. Male Wistar rats selectively bred for high innate anxiety are characterized by elevated ACTH and corticosterone responses to an adaptation of the combined DEX/CRH test, and treatment with an antagonist against the vasopressin receptor 1 normalized the HPA axis hyperactivity in these animals [65]. Several other findings support central AVP overexpression in these animals [66], which is caused by a genetic variation in the promoter region of the AVP gene [67].

In addition, the activating effect of lithium on the HPA system might be mediated by direct intracellular effects of lithium on pituitary cells [31], [32] and by the induction of gene expression [68].

Interestingly, when viewed separately with regard to responders and non-responders in the present study, the significant rise in the post-CRH ACTH and cortisol parameters under lithium treatment is only present in those who responded to the therapy. Most antidepressants, as tricyclic antidepressants [69], [70], [71], [72], [73], [74], [75] and SSRIs [76], [77], [78] seem to lead to a blunting of the HPA system response. Predominantly this is interpreted as a normalisation of the pathologically-activated HPA system in depressed subjects and seen as a possible way in which antidepressants exhibit their clinical effects [9], [41], [52], [79], [80]. However, as results in recent years have shown, the situation has become even more complex [9], [10], [58], [81]. In the case of lithium treatment, the stimulation of the HPA axis at least does not hinder its antidepressive effects.

In addition, the influence of psychotropic drugs on the HPA system seems to be time-dependent: the inhibiting effect of some antidepressants on the ACTH and cortisol release seems to need a number of weeks of treatment, whereas the acute effects tend to result in stimulation of the hormone release, at least in healthy volunteers [8]. In contrast mirtazapine acutely inhibits HPA system activity [82], [83]. Lithium augmentation, as stated above, activates the HPA system, especially in responders [39], [42]. The results of the study presented here suggest that the interpretation of HPA axis markers in the case of lithium treatment is different due to its stimulating effects on AVP, which do not exist for antidepressant drugs. There is a rising awareness of a rather complex interaction between affective disorders, antidepressive drugs and the HPA system [8]. A better understanding of the molecular basis is needed to give a starting point for the development of new drug therapies with a new mode of action [9].

The study presented here has methodological strengths and shortcomings. One key strong point is the fact that the study participants were all drug-naive. Although the initial inclusion criteria required a drug free period of only two weeks, we were able to include only subjects without pharmacological pre-treatment for the depressive index episode. This excludes the influence of any preceding pharmacotherapy on the clinical or endocrinological results. The drop-out rate was extremely low (no patients dropped out, and only one patient declined the second DEX/CRH test). Diagnoses and depressive symptoms were monitored and validated using established standardised clinical interviews and rating scales. With regard to the neuroendocrinological evaluation, however complex, the best-established and validated test – the combined DEX/CRH test – was used. Lithium therapy can be considered as optimal provided that lithium serum levels are very frequently monitored and that lithium levels are rapidly achieved within the therapeutic range (0.5 to 1.0 mEq/L) despite a good tolerability.

As the most important limitation, one could mention the lack of a control group. However, in keeping with most studies with the DEX/CRH test, our study concentrated on comparing endocrinological parameters before the start of the treatment (right before the intake of the first lithium tablet) and the parameters four weeks after the start of treatment. Since no other systematic changes took place except the commencement of the lithium therapy, it is rather unlikely that the changes found in the HPA system might be due to factors other than the lithium therapy and the possible therapeutic effects induced by it. However, it cannot be excluded that other aspects such as the passage of time (4 weeks) or placebo effects might have had an impact on the endocrinological results. The absence of a control group means that the clinical efficacy of the lithium monotherapy found in our study cannot reliably be interpreted. However, this was not the objective of the study.

In conclusion, this is the first study on lithium monotherapy for acute unipolar depression in more than 20 years, and the first prospective study of lithium monotherapy to ever use the DEX/CRH test. Given the results of the study presented here, of our preceding study with the DEX/CRH test on lithium augmentation, of previous studies of the HPA status in depressed or healthy subjects and the results of the pre-clinical studies in animals and cell cultures, it can now be regarded as having been established that lithium has a stimulating effect on the human HPA system. This is presumably mainly due to an activation of central AVP.

The pronounced effects found in our study confirm the fundamental significance of the HPA system to the pathophysiology and the treatment of depressive disorders. Developing fundamentally new treatment approaches continues to be a challenge, given the fact that some approaches directly targeting the HPA system have failed in recent years [9], [10] and given that we are still rather far away from understanding the complex molecular mechanism underlying the regulation of the glucocorticoids in humans.

## Supporting Information

Protocol S1 **Trial protocol.**
(DOC)

Checklist S1 **CONSORT 2010 checklist.**
(DOC)
