# Supplementary material for: Lithium Monotherapy Increases ACTH and Cortisol Response in the Dex/CRH Test in Unipolar Depressed Subjects. A Study with 30 Treatment-Naive Patients
Source: PLoS One. 2011 Nov 23;6(11):e27613. doi: 10.1371/journal.pone.0027613 (PMC3223180; doi:10.1371/journal.pone.0027613)
Supplement: Protocol S1 — Trial protocol. (DOC) [file pone.0027613.s001.doc]

Neuroendokrinologische Wirkungen von Lithium und selektiv serotonergen Antidepressiva auf den Cortisol-Regelkreis beim Menschen

kurz: LiCo-Studie

###### **Prüfplan**

Das Studienzentrum ist die Klinik für Pschiatrie und Psychotherapie des Universitätsklinikums Carl Gustav Carus an der Technischen Universität Dresden.

**Gesamtleiter** der Studie: OA Dr. med. Tom Bschor.

**Zusammenfassung**

Lithium ist ein weit verbreitetes, seit über 50 Jahren erfolgreich in der Behandlung affektiver Erkrankungen (Depression, Manie) eingesetztes Pharmakon. Seine Wirkweise konnte jedoch bis heute nicht befriedigend geklärt werden. Für *Antidepressiva* wird aufgrund neuer Erkenntnisse eine Wirkung durch direkte Beeinflussung des Cortisol-Regelkreises (Hypothalamus-Hypophysen-Nebennieren-Achse [engl.: HPA axis]) vermutet. In eigenen Voruntersuchungen konnte die Arbeitsgruppe mit dem kombinierten Dexamethason/CRH-Test (Dex/CRH-Test) für eine Lithium*augmentation* eine deutliche Aktivierung der HPA-Achse nachweisen. Dies lässt eine mögliche Serotonin-vermittelte Stimulation der HPA-Achse hypothetisieren, was in der geplanten Studie geklärt werden soll. Hierzu sollen depressive Patienten monotherapeutisch mit Lithium behandelt und in gleicher Weise mit dem Dex/CRH-Test untersucht werden. In Teilprojekt III werden depressive Patienten monotherapeutisch mit einem SSRI behandelt und in gleicher Weise mit dem Dex/CRH-Test untersucht. Eine systematische Verlaufsstudie mit dem Dex/CRH-Test bei mit SSRI behandelten depressiven Patienten existiert bislang nicht. Dieser Test ist aufwendig, aber der sensitivste und spezifischste Test zur Untersuchung der HPA-Achse beim Menschen.

**Stand der Forschung**

Lithium wird seit seiner Erstbeschreibung als Psychopharmakon in der modernen Wissenschaft durch John Cade 1949 [Cade 1949] systematisch als Therapeutikum bei affektiven Erkrankungen (Manie und Depression) erforscht. Es besitzt ausgeprägte antimanische [Bowden et al. 1994] und phasenprophylaktische Eigenschaften bei mono- [Souza und Goodwin 1991] und bipolaren [Maj 2000] affektiven Erkrankungen. Ebenfalls gut belegt ist seine akut antidepressive Wirkung, sowohl in monotherapeutischer Anwendung [Adli et al. 1998, Souza und Goodwin 1991], als auch im Rahmen der Lithiumaugmentation (d. h. additiv zu einem allein nicht ausreichend wirksamen Antidepressivum) [Bauer und Döpfmer 1999]. Die Lithiumtherapie besitzt heute für die Behandlung der affektiven Erkrankungen eine einzigartige zentrale Stellung und ist aus der psychiatrischen Praxis nicht wegzudenken. Lithium besitzt anscheinend eine eigenständige antisuizidale Wirkung [Müller-Oerlinghausen et al. 1994, Felber 1995, Müller-Oerlinghausen und Berghöfer 1999, Tondo et al. 2001].

Trotz des über 50jährigen Einsatzes von Lithium in der psychiatrischen Therapie und einer umfassenden Beforschung sind die Wirkmechanismen dieser Substanz nicht hinreichend geklärt. Dies ist aber weiter das Ziel internationaler Forschungsanstrengungen, da von einem besseren Verständnis der Wirkweise dieses einzigartigen Medikamentes nicht nur ein besseres Verständnis der pathophysiologischen Prozesse bei den affektiven Erkrankungen, sondern auch die Ableitung weiterführender Therapien mit verbesserter Wirksamkeit oder verbessertem Nebenwirkungsprofil erhofft werden kann [Manji und Lenox 2000, Manji et al. 2001].

Aufgrund der vielfältigen Labor-, Tier- und Humanforschung ist gesichert, dass das Ion Lithium im Unterschied zu den meisten Pharmaka *intra*zellulär wirkt und hier in eine Vielzahl von Stoffwechselprozessen eingreift. So beeinflusst Lithium den zentralnervösen Dopamin- und Acetylcholinstoffwechsel (vermutlich über eine Interaktion mit Second-Messenger-Systemen), den cyclo-AMP-Gehalt, den Inositolstoffwechsel, die Proteinphosphorylierung, das cerebrale N-Acetylaspartat und den cerebralen Proteinkinase C-Gehalt [Wood and Goodwin 1987, van Calker et al. 1997, Manji et al. 2000, Manji et al. 2001].

Nicht ausreichend erforscht ist jedoch, zu welchen Folgewirkungen diese Beeinflussung des intrazellulären Stoffwechsels führt und wie diese in Zusammenhang mit den therapeutischen Effekten stehen. In letzter Zeit konnte unter anderem eine Veränderung der mRNA-Synthese für verschiedene Genprodukte durch Lithium im Tierversuch gezeigt werden [Wang et al. 1999].

Das am besten bezüglich der affektiven Erkrankungen erforschte biologische System ist der Hypothalamus-Hypophysen-Nebennierenrinden-Regelkreis (engl. *hypothalamic-pituitary-adrenocortical axis*, kurz *HPA axis*). Hier konnten konsistent in einer Vielzahl von Untersuchungen Veränderungen bei depressiven Patienten festgestellt werden, die sich überwiegend als rückbildungsfähig bei Abklingen der Depression zeigten (“*State*-”, nicht “*Trait*-Marker”) [Übersicht: Holsboer 2000]. So besteht im Mittelwertvergleich zu Gesunden bei depressiven Patienten ein Hyperkortisolismus [Gibbons 1964, Steckler et al. 1999]. Provokationstests der HPA-Achse sind in der Lage, sensitivere und spezifischere Befunde zu liefern. So zeigt der Dexamethason-Suppressionstest (DST) bei ca. 40 % der depressiven Patienten nicht die physiologisch zu erwartende Suppression der körpereigenen Cortisolproduktion nach Einnahme des synthetischen Kortikoids Dexamethason am Vorabend [Carroll et al. 1981, Holsboer 1983]. Im CRH-Stimulationstest hingegen zeigen depressiv Erkrankte eine abgeschwächte ACTH- und Cortisolantwort [Gold et al. 1986, Holsboer et al. 1986].

Der sensitivste und spezifischste Test zur Untersuchung der HPA-Achse bei psychiatrischen Patienten ist die Kombination der beiden letztgenannten Tests zum kombinierten Dexamethason/CRH-Test (Dex/CRH-Test) [Holsboer 2000]. Hier zeigen, wie vielfach repliziert werden konnte, Patienten während der depressiven Phase eine deutliche Überstimulierbarkeit. Es wird davon ausgegangen, dass es während einer Behandlung mit einem Antidepressivum zu einer Normalisierung der veränderten HPA-Achsen-Funktion kommt [Barden et al. 1995]. So konnte für eine Behandlung mit einem *trizyklischen* Antidepressivum eine Rückbildung der pathologischen Überstimulierbarkeit im kombinierten Dex/CRH-Test gezeigt werden [Heuser et al. 1996, Holsboer-Trachsler et al. 1994]. Systematische Untersuchungen mit dem kombinierten Dex/CRH-Test bezüglich Veränderungen der HPA-Achsen-Regulation durch Behandlung mit den heute am häufigsten eingesetzten *selektiv-serotonergen* Antidepressiva (SSRI), oder anderen neuen Antidepressiva der 3. Generation (Vorher–Nachher-Vergleich) wurden bislang jedoch nicht publiziert.

Aus diesen Befunden wurde die Hypothese einer pathologischen HPA-Achsen-Überaktivität mit einer gestörten Autoregulation dieses Systems durch eine beeinträchtigte negative Feedback-Hemmung als Bedingung für die Entstehung oder Aufrechterhaltung einer depressiven Episode abgeleitet und eine direkte Einwirkung bewährter Antidepressiva auf die HPA-Achse, zum Beispiel im Sinne einer Verbesserung des negativen Feedbacks, postuliert [Holsboer 2000, Steckler et al. 1999].

Aufgrund dieser Erkenntnislage stellte sich die Frage, ob Lithium, als ebenfalls antidepressiv wirksame Substanz, ebenso messbare Veränderungen der HPA-Achsen-Regulation bewirkt und ob hierüber möglicherweise ein Teil seiner Wirkungen erklärt werden kann. Veränderungen am Cortisol-Regelkreis sind angesichts der vielfältigen bekannten zellulären Effekte von Lithium leicht vorstellbar, bisher aber beim Menschen unzureichend untersucht. Die aufgeworfene Frage wird von der Arbeitsgruppe beforscht und soll mit dem hier geplanten Projekt weiter erhellt werden.

Verschiedene tier- und laborexperimentelle Untersuchungen zeigen bereits, dass Lithium deutlichen Einfluss auf die Corticoidregulation ausübt. Im Rattenexperiment zeigten Sugawara et al. [1988] einen Anstieg von *Serum*-ACTH, -Corticosteroiden und -Vasopressin, sowie eine Zunahme der CRH- und eine Abnahme der Vasopressin-Konzentration in der Hypophyse nach Lithiumapplikation. In Hypophysenzellkulturen steigerte Lithium ebenfalls die ACTH-Produktion [Zatz und Reisine 1985, Reisine und Zatz 1987, Sugawara et al. 1988].

Peiffer et al. [1991] wiesen bei der Ratte nach, dass Lithiumapplikation zu einer Zunahme der Glukocorticoidrezeptor-Messenger-RNA führt. Semba et al. [2000] bestätigten diese Ergebnisse auch für eine geringere Lithiumdosis und fanden eine Zunahme der mRNA insbesondere im Hippocampus und im Nucleus paraventricularis des Hypothalamus, während sie keine Vermehrung der Mineralocorticoidrezeptor-Messenger-RNA (MR-mRNA) fanden.

Die Erkenntnislage beim Menschen ist bislang unzureichend. Zwar wurde von Platman und Fieve [1968] bereits vor fast 35 Jahren ein unerwarteter Anstieg des Plasma-Cortisols bei elf bipolar affektiv erkrankten Patienten vier Tage nach Beginn einer Lithiummedikation festgestellt (“this increase is surprising”), jedoch ist dieser Befund schwer interpretierbar, weil sich die Patienten uneinheitlich in einer manischen, hypomanen oder depressiven Phase befanden. 1971 bestätigten sie ihre Befunde [Platman et al. 1971], jedoch waren wiederum aktueller affektiver Zustand der Patienten und auch Zeitpunkt der Cortisolbestimmung nach Lithiumbeginn zwischen den verschiedenen Versuchsteilnehmern uneinheitlich. Sachar et al. [1970] hingegen konnten bei remittierten bipolar erkrankten Patienten keine signifikante Veränderung des Serumcortisols durch Lithiumgabe feststellen. Nach *lang*fristiger Lithiumgabe (1 Jahr) hingegen wurde eine *Ab*nahme des mittleren Serumcortisols gefunden [Smigan und Perris 1984]. Halmi et al. [1972] fanden bei gesunden Kontrollprobanden eine Abnahme der natürlichen circadianen Schwankungen des Serumcortisols nach Beginn einer Lithiummedikation, insbesondere durch eine Verminderung des morgendlichen Cortisolpeaks.

Die Datenlage ist also uneinheitlich. In den letzten 18 Jahren wurden keine relevanten, am Menschen erhobenen Befunde zu den neuroendokrinologischen Lithiumeffekten publiziert. Insbesondere existieren keine Untersuchungen mit dem kombinierten Dex/CRH-Test, dem sensitivsten und spezifischsten Test zur Untersuchung der HPA-Achse bei psychiatrischen Patienten.

Auch ist unklar, welche Auswirkungen eine selektiv serotonerge antidepressive Medikation (z. B. mit einem SSRI) auf die HPA-Achse hat. Die bisherigen systematischen Untersuchungen wurden mit trizyklischen Antidepressiva (TZA), die neben einer serotonergen auch eine noradrenerge Wirkung und eine Beeinflussung zahlreicher weiterer Transmittersysteme bewirken, durchgeführt (Amitriptylin: Heuser et al. [1996], Trimipramin: Holsboer-Trachsler et al. [1994]). Die beschriebenen Effekte unter Lithiumgabe und die unten dargestellten Ergebnisse der Arbeitsgruppe, die eine Stimulation der HPA-Achse unter der Lithiumaugmentation zeigen, sowie weitere Befunde mit serotonergen Substanzen, z. B. mit dem 5-HT_1A_-Rezeptoragonisten Buspiron, dessen Applikation ebenfalls eine Aktivierung des Corticoid-Regelkreises beim Menschen zu bewirken scheint [Maes et al. 1996], lassen es erforderlich erscheinen, gezielt zu untersuchen, ob die mit TZA gewonnenen Ergebnisse auf selektiv serotonerge Antidepressiva übertragbar sind. Die Klärung dieser Frage ist Teil der geplanten Studie.

Eigene Vorarbeiten

Als Paradigma für eine besonders rasche und gut belegte akut-antidepressive Wirkung von Lithium untersuchte die Arbeitsgruppe die Auswirkungen einer Lithium*augmentation* auf die HPA-Achse. Die Lithiumaugmentation ist ein bewährtes Behandlungsverfahren in der Akutbehandlung depressiver Patienten, die auf eine adäquat durchgeführte, ausreichend lange Monotherapie mit einem Antidepressivum nicht respondierten. Hierbei wird durch Zugabe von Lithium zur kontinuierlich fortgeführten Antidepressiva-Gabe ein Ansprechen auf die medikamentöse Behandlung erzielt. Neun doppelblinde Studien haben die Wirksamkeit einer Lithiumaugmentation im Vergleich zu einer Plazeboaugmentation belegt (Metaanalyse: [Bauer & Döpfmer 1999]). Bei diesem Verfahren handelt es sich um das wissenschaftlich am besten belegte Verfahren bei Patienten mit majorer Depression, die auf einen Behandlungsversuch mit einem Antidepressivum nicht oder nur unzureichend ansprechen [Bauer et al. 2002]. In einer eigenen doppelblinden, placebokontrollierten Studie konnte die Arbeitsgruppe erstmals die Wirksamkeit der Lithiumaugmentation auch in der Erhaltungstherapie depressiver Episoden nachweisen [Bauer et al. 2000, Bschor et al., im Druck]. In einer weiteren Untersuchung konnte die Forschergruppe zeigen, dass stärker ausgeprägte depressive Syndrome eine größere Chance für ein Ansprechen auf die Lithiumaugmentation haben als schwächer ausgeprägte [Bschor et al. 2001].

In einer kürzlich abgeschlossenen Studie mit 30 akut depressiven, monopolaren Patienten untersuchte die Arbeitsgruppe die Auswirkungen einer Lithiumaugmentation auf die HPA-Achse mit dem kombinierten Dex/CRH-Test direkt vor und drei bis vier Wochen nach Beginn der Augmentation [Bschor et al. 2002].

Für akut-depressive Syndrome ist eine pathologische Veränderung der HPA-Achsen-Regulation und ihre Normalisierung unter (Antidepressiva-) Therapie am besten belegt, so dass hier ein Vergleich mit den Lithiumeffekten von besonderem Interesse war. Unerwarteter Weise zeigte sich eine deutlich signifikante *Zunahme* der ACTH- und Cortisol-Stimulierbarkeit im kombinierter Dex/CRH-Test unter Lithiumaugmentation. Sowohl der ACTH- und Cortisol-Peak, als auch der absolute Anstieg (Delta) und die Fläche unter der Kurve (AUC) stiegen nach der CRH-Stimulation signifikant an. Obwohl die Lithiumaugmentation klinisch gut antidepressiv wirksam war, zeigte sich also unter dieser Behandlung ein gegenteiliger Effekt von dem, was unter einer Behandlung mit trizyklischen Antidepressiva nachgewiesen werden konnte. Diese signifikante Zunahme der Hormonantwort im kombinierten Dex/CRH-Test war bei Respondern und Non-Respondern gleichermaßen nachweisbar, bei den Respondern auf die Lithiumaugmentation aber sogar besonders ausgeprägt [Bschor et al. 2002].

Für die Lithiumaugmentation lässt sich demnach kein geradliniger Zusammenhang von hoher (pathologisch erhöhter) Stimulierbarkeit im kombinierten Dex/CRH-Test und depressiver Psychopathologie bzw. klinischer Besserung und Rückgang der Stimulierbarkeit im Dex/CRH-Test aufstellen. Die dargelegten Vorbefunde lassen keine sicheren Rückschlüsse zu, welche Effekte für den aktivierenden Effekt der Lithiumaugmentation auf die HPA-Achse verantwortlich sind. Gut etabliert ist die pro-serotonerge Wirkung von Lithium, sowohl im Sinne einer gesteigerten Serotonin– (5-HT–)produktion als auch –freisetzung [Wood und Goodwin 1987]. Ebenfalls bekannt ist eine Aktivierung der HPA-Achse durch Serotonin, wie sie zum Beispiel über 5-HT_1_- und 5-HT_2_-Rezeptoren sowie in vivo und in vitro für Serotonin­agonisten gezeigt werden konnte. Serotonerge Projektionen ziehen vom Nucleus raphe dorsalis u. a. zum Nucleus paraventricularis des Hypothalamus, wo die CRH-Freisetzung gesteuert wird. Auch von der Amygdala wird die CRH-Sekretion vermutlich über serotonerge Bahnen zum Nucleus paraventricularis moduliert (Übersicht: [Whitnall 1993]). Eine serotonerg vermittelte Aktivierung der HPA-Achse durch Lithium ist daher vorstellbar.

In der erwähnten Studie der Arbeitsgruppe unterschieden sich Responder und Non-Responder signifikant in der Cortisol/ACTH-Ratio im Dex/CRH-Test *vor* Beginn der Lithiumaugmentation, die damit prädiktiven Wert bezüglich des erwarteten Behandlungserfolges aufweist. Die Cortisol/ACTH-Ratio ist ein Index für die Sensitivität der Nebennierenrinde [Holsboer et al. 1995] und war bei den Non-Respondern auf die anschließend folgende Lithiumaugmentation signifikant höher, was als Hinweis auf eine stärker chronifizierte Depression mit konsekutiver Nebennierenrindenhyperplasie und hieraus resultierender ACTH-Überempfindlichkeit interpretiert werden kann [Bschor et al., eingereicht].

Die von der Arbeitsgruppe mit dem kombinierter Dex/CRH-Test festgestellten Effekte einer Lithiumaugmentation auf die HPA-Achse sind bislang unentdeckt gewesen. Nach wie vor ungeklärt ist, ob es sich hierbei a) um einen bisher nicht bekannten direkten Effekt des Ions auf die HPA-Achse handelt, ob dieser b) nur im Zusammenhang mit einer depressiven Psychopathologie auftritt oder ob es sich hierbei c) um eine spezielle Wirkung der Lithium*augmentation*, die zwingend eine Vorbehandlung und anschließende Kombination mit einem Antidepressivum voraussetzt, handelt. Diese Fragen sollen mit den geplanten Untersuchungen geklärt werden.

Für die unter c) formulierte Annahme spricht, dass, obwohl alle 30 Patienten der erwähnten Studie vor Beginn der Lithiumaugmentation ein depressives Syndrom vom Schweregrad einer Majoren Depression nach DSM IV (SKID I-gesichert) mit deutlicher Ausprägung (im Mittel 20, mindestens 15 Punkte auf der Hamilton Rating Scale for Depression, 17 Item-Version) aufwiesen, die ACTH- und Cortisol-Antwort im kombinierten Dex/CRH-Test nur schwach und in ihrem Ausmaß eher gesunden Kontrollkollektiven vergleichbar war. Unter Lithiumaugmentation näherten sich die Resultate typisch depressiven Konstellationen an (obwohl knapp die Hälfte der Patienten klinisch gut auf die Behandlung respondiert hatte). Demnach ist eine typische HPA-Achsen-Dysregulation, die mutmaßlich vor Beginn der anti­depressiven Medikation bestanden hatte, möglicher Weise durch die bei allen Versuchsteilnehmern vorausgegangene (wenngleich klinisch unzureichend wirksame) Antidepressiva-Behandlung normalisiert worden. Die Augmentation mit Lithium entfaltet hiernach erst auf dem Boden dieser zweifach (durch das depressive Syndrom und durch die Antidepressiva-Behandlung) veränderten HPA-Achsen-Steuerung den beobachteten Effekt auf die HPA-Achse. Die Aktivierung der HPA-Achse könnte hierbei im Sinne einer Stressreaktion auf die Lithiumgabe [Platman und Fieve 1968] verstanden werden.

*Hypothesen*

Auf der Basis der vorhandenen grundlagenwissenschaftlichen Erkenntnisse sind verschiedene Hypothesen zur Erklärung der Effekte der Lithiumaugmentation im kombinierten Dex/CRH-Test formulierbar, die in dem vorliegenden Antrag geprüft werden sollen:

*Die Zunahme der ACTH- und Cortisol-Stimulierbarkeit im kombinierten DEX/CRH Test unter Lithiumaugmentation resultiert aus einer*

1. *direkten serotonergen Wirkung des Ions Lithium, unabhängig von depressiver Psychopathologie und dem Einsatz als Augmentativum.*
2. *direkten, nicht-serotonerg vermittelten Lithiumwirkung, unabhängig von depressiver Psychopathologie und Einsatz als Augmentativum.*
3. *speziellen neuroendokrinologischen Wirkung der Augmentation, abhängig von einer ausreichend langen Vorbehandlung mit einem Antidepressivum.*

Studienablauf

*Patienten*

In Teilprojekt I und II werden Patienten mit einem behandlungsbedürftigen, akuten depressiven Syndrom vom Schweregrad einer Major Depression (DSM IV), das im Rahmen einer depressiven Einzelepisode oder im Rahmen eines monopolaren Verlaufs auftritt, eingeschlossen. *Bipolar* erkrankte Patienten werden *nicht* eingeschlossen. Die Studienteilnehmer sollen aus den Patienten der Klinik und Poliklinik für Psychiatrie und Psychotherapie der Technischen Universität Dresden rekrutiert werden; es sollen ambulant und stationär behandelte Patienten eingeschlossen werden. Es gelten folgende

*Einschlusskriterien:*

- Patienten beiderlei Geschlechts. Alter: 18-75 Jahre.
- Diagnose einer akuten depressiven Episode vom Schweregrad einer Major Depression nach DSM IV, diagnostiziert mit dem SKID IV.
- Schwere des depressiven Syndroms von mindestens 15 Punkten auf der Hamilton Rating Scale for Depression (HAM-D, 21 Item-Version).
- Klinische Entscheidung für eine antidepressive Behandlung unabhängig von der Studie.
- Schriftliches und mündliches Einverständnis nach eingehender schriftlicher und mündlicher Aufklärung.
- Einwilligungsfähigkeit.

*und Ausschlusskriterien:*

- manische oder hypomanische Episode in der Vorgeschichte (bipolare Erkrankung).
- psychotrope Medikation bei Studieneinschluss oder während einer Woche vor Studienbeginn (gfls. einwöchige Auswaschphase vor Studienbeginn). *Erlaubt* ist vor und während der Studie eine Benzodiazepinmedikation bis 20mg Diazepam-Äquivalent täglich.
- Schwere internistische Erkrankungen sowie Erkrankungen, die eine Lithium- bzw. eine SSRI-Medikation verbieten oder die Durchführung eines kombinierten Dex/CRH-Tests nicht sinnvoll auswertbar machen (z. B. schwere Nierenfunktionsstörungen, schwere Herz-Kreislauf­erkrankungen, Störungen der Gluko- oder Mineralocorticoidregulation).
- Schwangerschaft, Stillen oder unsichere Kontrazeption.
- Weitere Achse I Diagnosen (DSM IV) neben der affektiven Erkrankung
- Abhängigkeitserkrankungen (diagnostische Operationalisierung ebenfalls mit SKID IV).

*Untersuchungen vor Studienbeginn (Tag –3 bis –1)*

- SKID IV-Interview zur Diagnosebestätigung
- Depressions-Schweregrad mittels HAM-D (21 Item-Version)
- CGI (Clinical Global Ipression Scale [Guy 1976]): Schweregrad
- BDI (Beck Depression Inventory [Beck et al. 1961])
- Erfassung folgender Covariablen:
- Alter
- Geschlecht
- psychiatrische Komorbidität (Achse II-Diagnose)
- Dauer und Behandlung der Indexepisode bis Studieneinschluss
- Anzahl früherer Phasen
- Alter bei Erstmanifestation
- Anzahl früherer psychiatrischer Hospitalisationen
- internistische Erkrankungen
- Ausführliche internistische und neurologische körperliche Untersuchung
- Körpertemperatur, RR, EKG
- Routine-Laboruntersuchung (Differentialblutbild, Elektrolyte, Retentionswerte, Transaminasen, Alkalische Phosphatase, Nüchtern-Glucose, Schilddrüsenhormone T_3_, T_4_ und TSH, BSG)
- Schwangerschaftstest (β-HCG)

*Studienablauf*

***Tag 0***

**1. Dex/CRH-Test**

***abends:***

**Beginn Li bzw.**

***morgens Tag 1:***

**Beginn Citalopram**

***Tag 28***

**2. Dex/CRH-Test**

***Tag –3 bis –1***

**Eingangs-**

**untersuchungen**

|  |  |  |  |  |  |  |  |  |  |  |  |  |  |  |  |  |  |  |  |  |  |  |  |  |  |  |  |  |  | Tage |
| --- | --- | --- | --- | --- | --- | --- | --- | --- | --- | --- | --- | --- | --- | --- | --- | --- | --- | --- | --- | --- | --- | --- | --- | --- | --- | --- | --- | --- | --- | --- |
| –3 bis –1 | 0 | 1 |  | 3 |  |  |  | 7 |  |  |  |  |  |  | 14 |  |  |  |  |  |  | 21 |  |  |  |  |  |  | 28 |  |

**Li-Ktrl.**

**Li-Ktrl.,**

**HAM-D, CGI, BDI**

**Li-Ktrl.,**

**HAM-D, CGI, BDI**

**Li-Ktrl.**

**HAM-D, CGI, BDI**

**Li-Ktrl.,**

**HAM-D, CGI, BDI**

**Lithium /
Citalopram**

Li: Lithium

Li-Ktrl.: Kontrolle des 12 Std.-Lithium-Serumspiegels und Lithium-typischer Nebenwirkungen (nur Teilprojekt I)

HAM-D: Hamilton Rating Scale for Depression, 21 Item-Version; CGI: Clinical Global Impression Scale;
BDI: Beck Depression Inventory; Dex/CRH-Test: kombinierter Dexamethason/CRH-Test

Die Studie erstreckt sich pro Proband über vier Wochen. An den Tagen –3 bis –1 erfolgen die Kontrolle der Ein- und Ausschlusskriterien und die o. g. Untersuchungen. Außerdem wird der Proband umfangreich über die Besonderheiten einer Lithium- bzw. Citaloprammedikation und über Dinge, die er beachten muss, aufgeklärt. An den Tagen 0 und 28 erfolgt je ein kombinierter Dex/CRH-Test (Beschreibung: s. u.).

Die eingeschlossenen Patienten werden über vier Wochen monotherapeutisch antidepressiv behandelt, und zwar die Patienten in Teilprojekt I mit Lithium und die Patienten aus Teilprojekt II mit Citalopram. Erlaubt ist eine Begleitmedikation mit Benzodiazepinen bis zu einer täglichen Dosis von 20mg Diazepamäquivalent. Eine randomisierte Zuteilung der Patienten auf Teilprojekt II und III oder eine verblindete Medikation ist *nicht* vorgesehen, da die Studie nicht einen klinischen Wirksamkeitsvergleich zwischen Lithium und dem SSRI bezweckt.

Die Lithiummedikation beginnt am Tag 0 um 20.00h, *nach* dem ersten Dex/CRH-Test mit einer Tablette 12,2 mmol Lithium und wird ab Tag 1 mit zweimal täglich (8.00h und 20.00h) 12,2 mmol Lithium fortgesetzt. An den Tagen 3, 7, 14 und 21 erfolgen morgendliche Kontrollen des Lithium-Serumspiegels (12 Stunden nach der letzten Lithiumeinnahme) und die Erfassung Lithium-typischer Nebenwirkungen. Nach diesen Spiegeln wird die tägliche Lithium­dosis individuell adaptiert mit dem Ziel, einen Serumspiegel zwischen 0,6 und 0,8 mmol/l einzustellen. Zu Dokumentationszwecken wird der Lithium-Serumspiegel auch am Tag 28 bestimmt. Die Citalopramdosis für die Patienten im Teilprojekt II beträgt 20 mg täglich in der ersten Woche und ab dann 40mg täglich, kann bei unzureichender Verträglichkeit aber wieder auf 20 mg täglich gesenkt werden (Einmalgabe am Morgen).

Am Tag 0, vor Beginn der antidepressiven Behandlung und an Tag 28 wird der kombinierter Dex/CRH-Test (s. u.) durchgeführt. Direkt vor Beginn der Medikation und dann alle 7 Tage (s. Graphik) wird zur Beurteilung der Response der HAM-D und der CGI („Schweregrad“, ab der 2. Messung auch „Veränderung“) sowie als Selbstbeurteilungsskala das Beck-Depression Inventory (BDI) erhoben. Als Response wird eine Reduktion des HAM-D um mindestens 50% während des vierwöchigen Studienzeitraumes definiert. Die Response wird erhoben, um mögliche Zusammenhänge zwischen Veränderungen der HPA-Achsen-Regulation und einem Ansprechen auf die Behandlung feststellen zu können. Nach Ende der Studie wird der Patient nach ärztlichem Ermessen weiter behandelt, wobei bei Respondern eine mindestens sechsmonatige Fortführung der Medikation empfohlen wird. Bei Non-Respondern kommt ein Umsetzen auf ein anderes Antidepressivum oder eine Kombination von Lithium mit einem Antidepressivum in Betracht. Im Falle des Umsetzens von Patienten aus Teilprojekt II wird eine ausschleichende Beendigung der Lithiummedikation empfohlen.

*Der kombinierte Dex/CRH-Test*

Der kombinierte Dex/CRH-Test wird entsprechend der etablierten Vorgehensweise durchgeführt [Heuser et al. 1994, Zobel et al. 2001].

***Vortag***

23.00h orale Einnahme von 1,5 mg Dexamethason.

Durch die Gabe dieses synthetischen Kortikoids wird die körpereigene Cortisolproduktion supprimiert.

***Untersuchungstag***

- 12.00 Uhr standardisiertes Mittagessen
- 14.00 Uhr Legen einer Venenverweilkanüle in eine Unterarm-/Cubitalvene
- 15.00 Uhr Blutentnahme
- 15.02 Uhr 100μg CRH i.v.
- 15.30, 15.45, 16.00, 16.15, 16.30 Uhr weitere Blutentnahmen

Zur Reduktion stressbedingter Beeinflussung der Ergebnisse muss der Dex/CRH-Test unter standardisierten Bedingungen und unter möglichst ruhigen Umgebungsbedingungen für den Probanden stattfinden. Verboten ist daher körperlich, kognitiv oder emotional belastende Aktivität am Testtag (bis 16.30h). Während der Untersuchung selbst sitzt der Proband in einem Ruhesessel. Die Blutentnahmen erfolgen mit der sogenannten “Wall Through Technik", bei der von der Venenverweilkanüle eine Schlauchverlängerung durch eine kleine Öffnung in der Wand in einen Nebenraum führt, so dass der Patienten durch die Vorgänge der Blutentnahme und Injektion nicht irritiert wird. Der venöse Zugang wird zwischen den Blutentnahmen durch eine Infusomat-gesteuerte heparinhaltige Infusion (200IE Heparin in 500ml NaCl 0,9%, 80ml/h) offen gehalten.

Aus allen Blutproben (je 10ml) wird ACTH und Cortisol bestimmt. Alle Röhrchen sind mit 150μl Trasilol und 250μl EDTA vorbestückt. Wegen der Unbeständigkeit von ACTH müssen die Blutentnahmen in vorgekühlte Röhrchen auf Eis erfolgen und sofort kühlzentrifugiert werden (4°C, 4000 U, 7 Min.). Der Serumanteil wird sofort abpipettiert und sofort bei –80°C eingefroren.

Die Laboranalysen auf ACTH und Cortisol werden nach Ende der gesamten Studie in einem gemeinsamen Ansatz durchgeführt. Hierfür ist eine Kooperation mit dem Labor des Max-Planck-Instituts für Psychiatrie in München (Laborleiter: Dr. Manfred Uhr) vereinbart, das bereits die Analysen für die o. g. Studie der Arbeitsgruppe durchgeführt hat. Dieses Labor verfügt über die größte Erfahrung in der Laboranalyse des Dex/CRH-Tests in Deutschland.

##### Statistische Auswertung

Die statistische Auswertung erfolgt ebenfalls in Kooperation mit dem Max-Planck-Institut für Psychiatrie in München (Dr. Marcus Ising). Diese Kooperation hatte sich bereits bei der o. g. Studie der Arbeitsgruppe bewährt.

*Fallzahlabschätzung:* Für die Fallzahlabschätzung wird auf die Ergebnisse der mehrfach erwähnten Vorstudie zur Lithiumaugmentation Bezug genommen. Die Fallzahlberechnung für diese Studie hatte in Anlehnung an die Arbeit von Holsboer et al. [1995] folgende Annahmen zugrunde gelegt: Erwartete Differenz des Cortisol-Peaks 203 nmol/l, Standardabweichung: 125 nmol/l, Power: 80%, Signifikanzniveau: 0,05. Damit hatte sich eine Mindestfallzahl von 15 Teilnehmern pro Gruppe für den Responder–Non-Responder–Vergleich errechnet. In der Vorstudie hatte sich in der Gesamtgruppe der 24 nachuntersuchten Teilnehmer ein deutlich signifikanter Effekt durch die Lithiumaugmentation im kombinierten Dex/CRH-Test gezeigt. In der hier zur Beantragung vorgestellten Studie sollen daher pro Teilprojekt 30 Versuchsteilnehmer untersucht werden.

*Outcome-Analyse:* Es werden für alle Dex/CRH-Tests, jeweils für ACTH und Cortisol getrennt, die Peak-, die Delta und die AUC- (area under the curve) Werte bestimmt. Hauptzielparameter der Studie sind die Veränderungen dieser endokrinologischen Werte im Behandlungsverlauf (jeweils innerhalb eines Teilprojektes). Diese werden mit dem Wilcoxon matched pairs Test verglichen. Vergleiche zwischen Respondern und Non-Respondern (Teilprojekt II und III) werden mit dem Mann-Whitney U Test, bzw. für dichotome Variablen mit Fisher’s exact Test durchgeführt. Das Signifikanzniveau wird auf p < 0,05 festgelegt.

Literatur

Adli M, Bschor T, Canata B, Döpfmer S, Bauer M (1998) Lithium in der Behandlung der akuten Depression. Fortschr Neurol Psychiat 66:435-441

Barden N, Reul JM, Holsboer F (1995) Do antidepressants stabilize mood through actions on the hypothalamic-pituitary-adrenocortical system? Trends Neurosci 18:6-11

Bauer M, Bschor T, Kunz D, Berghöfer A, Ströhle A, Müller-Oerlinghausen B (2000) Double-blind, placebo-controlled trial of the use of lithium to augment antidepressant medication in continuation treatment of unipolar major depression. Am J Psychiatry 157:1429-1435

Bauer M, Döpfmer S (1999) Lithium augmentation in treatment-resistant depression - A meta-analysis of placebo-controlled studies. J Clin Psychopharmacology 19:427-434

Bauer M, Whybrow PC, Angst J, Versiani M, Möller HJ (2002) World Federation of Societies of Biological Psychiatry (WFSBP) Guidelines for biological treatment of unipolar depressive disorders. Part 1. Acute and continuation treatment of major depressive disorder. World J Biol Psychiatr 3:5-43

Beck AT, Ward CH, Mendelson M, Mock J, Erbaugh J (1961) An inventory for measuring depression. Arch Gen Psychitry 4: 561-571

Bowden CL, Brugger AM, Swann AC, Calabrese JR, Janicak PG, Petty F, Dilsaver SC, Davis JM, Rush AJ, Small JG, Garza-Treviño ES, Risch C, Goodnick PJ, Morris DD (1994) Efficacy of divalproex vs lithium and placebo in the treatment of mania. The Depakote Mania Study Group. JAMA 271:918-924

Bschor T, Adli M, Baethge C, Eichmann U, Ising M, Uhr M, Modell S, Künzel H, Müller-Oerlinghausen B, Bauer M (2002) Lithium augmentation increases the ACTH and cortisol response in the combined DEX/CRH test in unipolar major depression. Neuropsychopharmacology, Papierversion im Druck, Online-Publikation am 4.3.2002 unter www.acnp.org/citations/Npp030402258

Bschor T, Baethge C, Adli M, Eichmann U, Ising M, Uhr M, Modell S, Künzel H, Müller-Oerlinghausen B, Bauer M. Association Between Response to Lithium Augmentation and the Combined DEX/CRH Test in Major Depressive Disorder, eingereicht

Bschor T, Berghöfer A, Ströhle A, Kunz D, Adli M, Müller-Oerlinghausen B, Bauer M. How long should the lithium augmentation strategy be maintained? A 1-year follow up of a placebo-controlled study in unipolar refractory major depression. J Clin Psychopharmacol, im Druck

Bschor T, Canata B, Müller-Oerlinghausen B, Bauer M (2001) Predictors of response to lithium augmentation in tricyclic antidepressant-resistant depression. J Affect Disord 64:261-265

Cade JFJ (1949) Lithium salts in the treatment of psychotic-excitement. Med J Aust 36:349-352

Carroll BJ, Feinberg M, Greden JF, Tarika J, Albala AA, Haskett RF, James NM, Kronfol Z, Lohr N, Steiner M, de Vigne JP, Young E (1981) A specific laboratory test for the diagnosis of melancholia. Standardization, validation, and clinical utility. Arch Gen Psychiatry 38:15-22

Felber W (1995) Suizide und Parasuizide bei Lithiumpatienten in der lithiumwirksamen und lithiumunwirksamen Zeit. In: Wolfersdorf M, Felber W (Hg.) Suizidologie Bd. 2. Regensburg, S. Roderer, S. 205-214

Gibbons JL (1964) Cortisol secretion rate in depressive illness. Arch Gen Psychiatry 10:572-575

Gold PW, Loriaux DL, Roy A, Kling MA, Calabrese JR, Kellner CH, Nieman LK, Post RM, Pickar D, Gallucci W, Avgerinos P, Paul S, Oldfield EH, Cutler GB Jr, Chrousos GP (1986) Response to corticotropin-releasing hormone in the hypercortisolism of depression and Cushing’s disease. N Engl J Med 314:1329-1335

Guy W (1976) ECDEU assessment manual for psychopharmacology. US Department of Health, Education and Welfare, Public Health Service, Alcohol, Drug Abuse and Mental Health Administration. Rockville, Maryland

Halmi KA, Noyes R Jr, Millard SA (1972) Effect of lithium on plasma cortisol and adrenal response to adrenocorticotropin in man. Clin Pharmacol Ther 13:699-703

Hamilton M (1960) A rating scale for depression. J Neurol Neurosurg Psychiatry 23:6-62

Heuser I, Yassouridis A, Holsboer F (1994) The combined dexamethasone/CRH test: a refined laboratory test for psychiatric disorders. J Psychiatr Res 28:341-356

Heuser IJE, Schweiger U, Gotthardt U, Schmider J, Lammers CH, Dettling M, Yassouridis A, Holsboer F (1996) Pituitary-adrenal-system regulation and psychopathology during amitriptyline treatment in elderly depressed patients and normal comparison subjects. Am J Psychiatry 153:93-99

Holsboer F (1983) Prediction of clinical course by dexamethasone suppression test (DST) respons in depressed patients – physiological and clinical construct validity of the DST. Pharmacopsychiatria 16:186-191

Holsboer F (2000) The corticosteroid receptor hypothesis of depression. Neuropsychopharmacol 23:477-501

Holsboer F, Gerken A, von Bardeleben U, Grimm W, Beyer H, Müller OA, Stalla GK (1986) Human corticotropin-releasing hormone (CRH) in patients with depression, alcoholism and panic disorder. Biol Psychiatry 21:601-611

Holsboer, F, Lauer, Ch J, Schreiber, W, Krieg, JC (1995) Altered hypothalamic-pituitary-adrenocortical regulation in healthy subjects at high familial risk for affective disorders. Neuroendocrinology 62:340-347

Holsboer-Trachsler E, Hemmeter U, Hatzinger M, Seifritz E, Gerhard U, Hobi V (1994) Sleep deprivation and bright light as potential augmenters of antidepressant drug treatment--neurobiological and psychometric assessment of course. J Psychiatr Res 28:381-399

Maes M, Van Gastel A, Meltzer HY, Cosyns P, Blockx P, Desnyder R (1996) Acute administration of buspirone increases the escape of hypothalamic-pituitary-adrenal-axis hormones from suppression by dexamethasone in depression. Psychoneuroendocrinology 21:67-81

Maj M (2000) The impact of lithium prophylaxis on the course of bipolar disorder: a review of the research evidence. Bipolar Disord 2:93-101

Manji HK, Lenox RH (2000) Signaling: cellular insights into the pathophysiology of bipolar disorder. Biol Psychiatry 48:518-530

Manji HK, Moore GJ, Chen G (2000) Clinical and preclinical evidence for the neurotrophic effects of mood stabilizers: implications for the pathophysiology and treatment of manic-depressive illness. Biol Psychiatry 48:740-754

Manji HK, Moore GJ, Chen G (2001) Bipolar disorder: leads from the molecular and cellular mechanisms of action of mood stabilizers. Br J Psychiatry 178 (Suppl 41):S107-119

Müller-Oerlinghausen B, Berghöfer A (1999) Antidepressants and suicidal risk. J Clin Psychiatry 60 (Suppl 2):94-99

Müller-Oerlinghausen B, Wolf T, Ahrens B, Schou M, Grof E, Grof P, Lenz G, Simhandl C, Thau K, Wolf R (1994) Mortality during initial and during later lithium treatment. A collaborative study by the International Group for the Study of Lithium-treated Patients. Acta Psychiatr Scand 90:295-297

Peiffer A, Veilleux S, Barden N (1991) Antidepressant and other centrally acting drugs regulate glucocorticoid receptor messenger RNA levels in rat brain. Psychoneuroendocrinology 16:505-515

Platman SR, Fieve RR (1968) Lithium carbonate and plasma cortisol response in the affective disorders. Arch Gen Psychiatry 18:591-594

Platman SR, Hilton JG, Koss MC, Kelly WG (1971) Production of cortisol in patients with manic-depressive psychosis treated with lithium carbonate. Dis Nerv Syst 32:542-544

Reisine T, Zatz M (1987) Interactions among lithium, calcium, diacylglycerides, and phorbol esters in the regulation of adrenocorticotropin hormone release from AtT-20 cells. J Neurochem 49:884-889

Sachar EJ, Hellman L, Kream J, Fukushima DK, Gallagher TF (1970) Effect of Lithium-carbonate therapy on adrenocortical activity. Arch Gen Psychiatry 22:304-307

Semba J, Watanabe H, Suhara T, Akanuma N (2000) Chronic lithium chloride injection increases glucocorticoid receptor but not mineralocorticoid receptor mRNA expression in rat brain. Neurosci Res 38:313-319

Smigan L, Perris C (1984) Cortisol changes in long-term lithium therapy. Neuropsychobiology 11:219-223

Souza FG, Goodwin GM (1991) Lithium treatment and prophylaxis in unipolar depression: a meta-analysis. Br J Psychiatry 158:666-675

Statistisches Bundesamt (1998) Gesundheitsbericht für Deutschland. 5.15 Depressionen. Statistisches Bundesamt, Wiesbaden

Steckler T, Holsboer F, Reul JM (1999) Glucocorticoids and depression. Baillieres Best Pract Res Clin Endocrinol Metab 13:597-614

Sugawara M, Hashimoto K, Hattori T, Takao T, Suemaru S, Ota Z (1988) Effects of lithium on the hypothalamo-pituitary-adrenal axis. Endocrinol Jpn 35:655-663

Tondo L, Hennen J, Baldessarini RJ (2001) Lower suicide risk with long-term lithium treatment in major affective illness: a meta-analysis. Acta Psychiatr Scand 104:163-172

van Calker D, Walden J, Berger M (1997) Effekte von Lithiumionen auf Neurotransmitter und sekundäre Botenstoffe. In: Müller-Oerlinghausen B, Greil W, Berghöfer A (Hg.) Die Lithiumtherapie. Berlin, Springer, S. 35-60

von Bardeleben U, Holsboer F (1991) Effect of age on the cortisol response to human corticotropin-releasing hormone in depressed patients pretreated with dexamethasone. Biol Psychiatry 29:1042-1050

Wang JF, Chen B, Young LT (1999) Identification of a novel lithium regulated gene in rat brain. Molecular Brain Research 70:66-73

Whitnall MH (1993) Regulation of the hypothalamic corticotropin-releasing hormone neurosecretory system. Progress in Neurobiology 40: 573-629

Wittchen, HU, Wunderlich, U, Gruschwitz, S, Zaudig, M (1997) SKID-I. Strukturiertes Klinisches Interview für DSM-IV. Achse I: Psychische Störungen. Göttingen, Hogrefe

Wood AJ, Goodwin GM (1987) A review of the biochemical and neuropharmacological actions of lithium. Psychol Med 17:579-600

Zatz M, Reisine TD (1985) Lithium induces corticotropin secretion and desensitization in cultured anterior pituitary cells. Proc Natl Acad Sci USA 82:1286-1290

Zobel AW, Nickel T, Sonntag A, Uhr M, Holsboer F, Ising M (2001) Cortisol response in the combined dexamethasone/CRH test as predictor of relapse in patients with remitted depression. a prospective study. J Psychiatr Res 35:83-94
